# The evolving role of nursing informatics in the era of artificial intelligence

**DOI:** 10.1111/inr.13084

**Published:** 2025-01-10

**Authors:** Abdulqadir J. Nashwan, JC A. Cabrega, Mutaz I. Othman, Mahmoud Abdelwahab Khedr, Yasmine M. Osman, Ayman Mohamed El‐Ashry, Rami Naif, Ahmad A. Mousa

**Affiliations:** ^1^ Assistant Executive Director of Nursing and Midwifery Research Nursing and Midwifery Research Department Hamad Medical Corporation Doha Qatar; ^2^ Informatics Nurse, Nursing Informatics Department Hamad Medical Corporation Doha Qatar; ^3^ Charge Nurse, Nursing Department Hamad Medical Corporation Doha Qatar; ^4^ Faculty of Nursing, Alexandria University, Alexandria, Egypt Alexandria University Alexandria Egypt; ^5^ Department of Obstetrics and Gynaecology Nursing, Faculty of Nursing Zagazig University Zagazig Egypt; ^6^ Faculty of Nursing Alexandria University Alexandria Egypt; ^7^ Senior Manager Consulting Services, Oracle Dubai UAE; ^8^ Nursing Researcher & Lecturer, Edith Cowan University Western Australia Australia

**Keywords:** AI literacy, artificial intelligence, clinical decision support, data privacy, ethics, health policy, healthcare, nursing informatics, nursing practice, patient care

## Abstract

**Aim:**

This narrative review explores the integration of artificial intelligence (AI) into nursing informatics and examines its impact on nursing practice, healthcare delivery, education, and policy.

**Background:**

Nursing informatics, which merges nursing science with information management and communication technologies, is crucial in modern healthcare. The emergence of AI presents opportunities to improve diagnostics, treatment, and healthcare resource management. However, integrating AI into nursing practice also brings challenges, including ethical concerns and the need for specialized training.

**Sources of evidence:**

A comprehensive literature search was conducted from January 2013 to December 2023 using databases like PubMed, Google Scholar, and Scopus. Articles were selected based on their relevance to AI's role in nursing informatics, particularly in enhancing patient care and healthcare efficiency.

**Discussion:**

AI significantly enhances nursing practice by improving diagnostic accuracy, optimizing care plans, and supporting resource allocation. However, its adoption raises ethical issues, such as data privacy concerns and biases within AI algorithms. Ensuring that nurses are adequately trained in AI technologies is essential for safe and effective integration.

**Implications for nursing practice and policy:**

Policymakers should promote AI literacy programs for healthcare professionals and develop ethical guidelines to govern the use of AI in healthcare. This will ensure that AI tools are implemented responsibly, protecting patient rights and enhancing healthcare outcomes.

**Conclusion:**

AI offers promising advancements in nursing informatics, leading to more efficient patient care and improved decision‐making. Nonetheless, overcoming ethical challenges and ensuring AI literacy among nurses are critical steps for successful implementation.

## INTRODUCTION

### Definition and role of nursing informatics

Nursing informatics, as defined by the American Nurses Association (ANA), integrates nursing science, computer science, and information science to manage and communicate data, information, knowledge, and wisdom in practice (American Nurses Association, 2022). It leverages technology to improve patient care. Nursing informatics competency integrates the knowledge, skills, and attitudes needed to perform informatics activities effectively. It focuses on leveraging technology to improve patient care and healthcare delivery. Nursing informatics facilitates the management of healthcare information systems, enabling nurses to make data‐driven decisions that enhance the quality of care. Furthermore, it supports developing and implementing technologies that assist clinical decision‐making, patient monitoring, and healthcare administration (Reid et al., 2021).

### Advancements in healthcare through AI

Since 2014, artificial intelligence (AI) has provided significant advancements in healthcare, potentially completely transforming the field by offering personalized, accurate, and innovative solutions (Badal et al., 2023). AI technology is increasingly used in healthcare and is expected to impact patient care and how healthcare professionals perform their work (Tursunbayeva & Renkema, 2023). AI is seen as a force in healthcare with the potential to bring about changes such as improving diagnosis and treatment, enhancing efficiency, optimizing resource allocation, and enhancing the quality of care, efficiency, and accuracy (Mohammed et al., 2022).

Nursing informatics is not limited to managing healthcare information systems. It has expanded to incorporate advanced technologies like AI, rapidly reshaping healthcare practices by offering personalized, efficient, and data‐driven solutions.

## PURPOSE OF THE REVIEW

This narrative review explores how AI is integrated into nursing informatics, tracing its origins and reviewing its application in healthcare. It discusses challenges, considerations, and ethical implications while highlighting its impact on nursing education and healthcare delivery.

## BACKGROUND

### Origins of nursing informatics

Nursing informatics can trace its roots back to Florence Nightingale, who utilized data to improve healthcare outcomes. The introduction of computers into healthcare in the 1950s further expanded the field (Vošner et al., 2020). In 1976, Scholes and Barber highlighted the potential of computer technology for nursing practice, education, and research (Scholes et al., 1983). During the 1980s, nursing informatics gained recognition with the increased accessibility of computers and other technology in the healthcare setting. ANA defined the field as integrating nursing science, computer science, and information science in identifying, collecting, and managing data and information to support nursing practice, administration, education, and research and to expand nursing knowledge (Kleib et al., 2021). Nursing informatics is critical to healthcare improvement, although insufficient informatics competency by nurses can result in suboptimal patient care (Jouparinejad et al., 2020).

### Early applications for informatics in nursing

In the 1980s, computer technology improved patient safety through automated dispensing cabinets that reduced medication errors. Hospital information systems (HIS) were developed, integrating tasks like admissions, discharges, and transfers (Collen & Miller, 2015).

### Advent of AI in healthcare

AI was introduced in 1955 but applied in healthcare in the 1970s with systems like INTERNIST‐I and CASNET model, which assisted in medical diagnoses (Bhikhubhai & Sharma, 2019; Stafie et al., 2023). AI has been shown to improve nursing management, quality, safety, and team communication, indicating its potential to enhance nursing practice and management (Chang et al., 2022).

### Integration of AI into nursing informatics

One of the key applications of AI in nursing informatics is clinical decision support systems (DSS) development. Over the past 10 years, AI‐based healthcare technologies have become increasingly prevalent, and their potential to enhance healthcare is quite promising (Kumar et al., 2023). Despite limited research on AI applications in nursing informatics, its potential to impact nursing practice is promising. Nurses, as frontline healthcare providers, are pivotal in AI integration. AI enables them to focus on direct patient care by automating data analysis, pattern recognition, and assisting in treatment planning (Ruksakulpiwat et al., 2024). AI systems enhance nursing informatics by improving decision‐making, patient monitoring, administrative tasks, and clinical documentation, enabling precise, individual‐based patient care. These advancements, in turn, increase their comprehensive capacity (Delaney et al., 2022).

## METHODOLOGY OF LITERATURE SELECTION

A comprehensive review was conducted to identify and summarize scientific literature on the role of nursing informatics in the era of AI in nursing. To ensure that there was a wide range of sources related to the changing role of nursing informatics in the era of AI, a comprehensive systematic search was conducted. We searched several databases, such as PubMed, Google Scholar, and Scopus. Boolean operators “AND” and “OR” were used to form different combinations of keywords to refine the search results by using terms pertinent to nursing, informatics, and AI: (“nurs*” OR “nursing ”OR “nurses ”OR “Healthcare”) AND (“Informatics” OR “Health informatics” OR “nursing informatics”) AND (“Artificial Intelligence” OR “AI” OR “machine learning” OR “neural networks” OR “deep learning” OR “natural language processing”). The review was not limited based on publication dates; however, preference was given to studies from January 2013 to December 2023 to capture the developments, including all papers in the English language. Initially, titles and abstracts were evaluated for relevance and adequacy to the set criteria, followed by examining the texts of selected articles to determine their rigor and contribution to the topic.

## CLINICAL DECISION SUPPORT SYSTEMS

AI‐powered CDSS have revolutionized nursing care by providing high‐quality, efficient care. AI‐CDSS analyzes patient data such as medical history, symptoms, and test results, helping clinicians make evidence‐based decisions and reducing medical errors (Ng et al., 2022; Sutton et al., 2020). A recent model using a long short‐term memory (LSTM) algorithm predicted home healthcare needs, significantly improving sensitivity and specificity compared with rule‐based systems (Juang et al., 2022). Another example is the Rothman Index, an AI‐derived score used in hospitals to predict patient deterioration and assist in timely interventions (Robert, 2019). This tool integrates data from electronic health systems (EHS) across various units and helps nurses prioritize care based on real‐time patient risk. Tools like the Rothman Index demonstrate the successful integration of predictive analytics into nursing practice through CDSS. AI algorithms support clinicians by processing large data sets, estimating diagnostic or prognostic outcomes, and automating clinical documentation. They streamline administrative duties, prioritize patient needs, and improve communication within healthcare teams, freeing nurses to focus on direct patient care. AI's ability to continuously assess patient data from various sources, such as wearable devices, helps nurses make informed, timely decisions, improving patient outcomes (Ng et al., 2022).

## AI AND PATIENT MONITORING

### Predictive analytics in nursing

AI‐powered predictive analytics have transformed patient monitoring by enabling nurses to proactively anticipate adverse events and manage patient care. Wearables, smart devices, and continuous monitoring systems allow real‐time data collection on vital signs, behaviors, and health conditions. AI algorithms analyze these data streams to predict health outcomes, such as readmissions, mortality, and complications like acute kidney injury (AKI) (Shaik et al., 2023). These tools enhance patient safety by providing timely alerts, allowing nurses to intervene before complications arise.

### AI for real‐time patient monitoring

AI‐based systems have significantly advanced real‐time patient monitoring. In intensive care units (ICUs), AI tools predict complications like sepsis and provide early warnings to healthcare teams. Syed et al. (2021) found that AI applications in ICUs are most frequently used for predicting readmissions, AKI, and mortality. AI‐powered monitoring systems notify nurses and other healthcare workers of abnormalities in patient status, enabling them to act promptly, which improves patient outcomes and optimizes healthcare resource allocation (Roski et al., 2019). This proactive approach reduces the strain on healthcare resources, making it easier to provide high‐quality, safe care to patients.

## CHALLENGES AND ETHICAL CONSIDERATIONS

### Ethical implications of AI in nursing

Integrating AI into nursing presents numerous ethical challenges that must be carefully considered. AI's potential to significantly impact nursing practices requires nurses to understand the development and implications of these technologies. Ethical concerns revolve around privacy, security, transparency, accountability, and maintaining the core values of nursing, such as empathy and human interaction (Ronquillo et al., 2021). As AI systems become more prominent in healthcare, ensuring they align with nursing's ethical foundations is paramount.

### Data privacy and security concerns

AI systems in healthcare rely heavily on large‐scale data collection, raising serious concerns about data privacy and security. With the increasing use of IoT‐based healthcare devices, patient data can be more vulnerable to breaches. Addressing these concerns involves creating transparent policies about using healthcare data, securing sensitive information, and overcoming technical obstacles related to big data management (Petersson et al., 2022). Regulatory frameworks and privacy standards are essential to protect patient information while enabling the advancement of AI technologies.

### Addressing the digital divide

The digital divide is another significant challenge to the equitable use of AI in nursing. The gap between those with access to advanced technologies and those without can lead to disparities in healthcare outcomes. Most AI applications in healthcare are concentrated in high‐income countries, with limited access to lower‐income regions. Efforts must be made to ensure that AI tools are accessible to all healthcare systems and communities, particularly those with limited technological infrastructure (Litchfield et al., 2021).

### Training and education in AI technologies

Adequate training and education are critical to ensuring nurses can effectively use AI technologies in clinical practice. AI‐based education tools offer personalized learning experiences, which could revolutionize nursing education by improving knowledge retention and practical application (Akgun & Greenhow, 2022). However, nursing schools must incorporate AI and nursing informatics into their curricula to prepare nurses for a tech‐driven future. Practical‐based courses and simulations will ensure that nurses acquire the necessary skills to integrate AI into patient care (Lukianets & Lukianets, 2023).

## CASE STUDIES AND REAL‐WORLD EXAMPLES

### Successful integration of AI in nursing

Real‐world applications of AI in nursing show its potential to transform patient care. One example is the Rothman index, an AI‐derived score that uses data from EHS to predict patient deterioration and help nurses prioritize care interventions (Keim‐Malpass et al., 2023). AI has also been successfully integrated into CDSS, with tools like the LSTM‐based model predicting healthcare needs based on patient data, significantly improving patient outcomes (Juang et al., 2022).

### Lessons learned from challenges

Despite successful integrations, AI adoption in nursing is challenging. Bias in AI algorithms and data quality concerns can lead to errors in patient care. AI systems rely heavily on the quality of data generated, and any flaws in data entry or collection can affect their performance. Nurses must validate the data fed into AI systems and combine AI‐generated recommendations with clinical judgment to mitigate these risks (O'Connor et al., 2023).

### Comparative analysis across healthcare systems

A systematic review conducted in 2022 found that AI‐based systems can significantly enhance early diagnosis, patient monitoring, and clinical decision‐making in nursing practice (Martinez‐Ortigosa et al., 2023). Another rapid review published in 2020 highlighted the predominance of AI applications in direct care support, care organization, and risk estimation, emphasizing AI's growing role in these areas (Seibert et al., 2021).

## DISCUSSION

AI and nursing informatics applications have already started to be part of daily nursing processes, such as virtual assistants and chatbots, clinical decision support apps, and robotics in nursing tasks such as medication delivery and repetitive procedures (Clancy, 2020). It is important to note that adopting AI in nursing will require collaboration between healthcare professionals, technologists, and policymakers to address challenges and ensure the ethical and responsible use of these technologies in patient care procedures and patient monitoring (Ronquillo et al., 2021). Future developments will likely focus on improving the interoperability of different healthcare systems, ensuring seamless integration of AI tools with existing electronic health records and HIS. This review provides valuable insights and recommendations for nurses looking to leverage the potential of nursing informatics and AI in their daily clinical activities, nursing education, and policies.

## FUTURE OF NURSING INFORMATICS IN THE AI ERA

### Predictions for AI advancements in nursing

The future of AI in nursing holds immense potential. AI will enhance disease diagnosis, treatment selection, and population health management. In particular, personalized medicine and AI‐driven clinical tools will enable nurses to provide more accurate, individualized care (Alowais et al., 2023). AI will also revolutionize mental health care, patient education, and nursing workflow, offering virtual health assistants and improved patient engagement tools (Booth et al., 2021).

### Role of nurses in AI development

Nurses play a vital role in developing and integrating AI technologies in healthcare. Nurses collaborate with data scientists and AI developers to ensure that AI applications are clinically relevant and patient‐centered. Nursing leaders must advocate for nurse involvement in AI technologies' design, implementation, and evaluation phases to maintain the profession's standards and ethical values (Matheny et al., 2019).

### Preparing the nursing workforce for AI

Preparing nurses for an AI‐driven future requires a strong focus on training and education. Programs like the Nurse Informatics Boot Camp offer practical AI and data analytics skills, preparing nurses to work alongside automated systems in clinical settings. Incorporating AI‐based simulations and hands‐on experiences into nursing curricula will ensure that the next generation of nurses is proficient in AI technologies and capable of leading healthcare innovations (Booth et al., 2021).

## RECOMMENDATIONS

### Recommendations for nursing practice

- Integrating AI into nursing practice can streamline workflows and improve patient outcomes by automating routine tasks and enhancing decision‐making through predictive analytics (Gopal et al., 2019).
- Collaboration between nurses, data scientists, and healthcare professionals is essential for successfully adopting AI tools. Effective communication and teamwork will be key to maximizing the benefits of AI in clinical practice.
- Nursing informatics and AI can directly impact patient care. Real‐world examples of successful implementations demonstrate improved diagnosis, treatment planning, and personalized care approaches.

### Recommendations for nursing education

- Nursing educators should integrate AI concepts and nursing informatics into the curriculum to prepare students for a tech‐driven healthcare environment.
- Providing hands‐on experiences with AI technologies and simulations will enable nursing students to develop practical skills for future practice (Božić, 2024).
- Familiarize nursing students with the fundamentals of AI by developing modules that explain AI applications in healthcare, from decision support systems to predictive analytics, ensuring students grasp the potential impact of AI on nursing roles.

## IMPLICATIONS FOR NURSING PRACTICE AND POLICY

### Shaping nursing policies for AI integration

Policymakers must recognize AI's transformative potential in nursing practice and develop policies that support its ethical and responsible integration (Cummins et al., 2021). Data privacy and security regulations must be strengthened to protect patient information while promoting innovation. Additionally, funding should be allocated to improve the technological infrastructure required for AI implementation in healthcare settings.

### Advocating ethical AI use in nursing

Nurses play a critical role in advocating for ethical AI use. They must ensure that AI systems uphold patient consent and transparency in data usage, addressing concerns about the misuse of patient information. Policies should promote clear communication regarding data collection, storage, and sharing, enabling patients to make informed decisions about their healthcare.

## CONCLUSION

Nursing informatics combines nursing science, information management, and communication technologies to promote well‐being. Integrating AI into nursing informatics is set to transform healthcare, improving patient outcomes through personalized care, enhanced decision‐making, and optimized workflows. However, data privacy, ethical considerations, and adequate training still need to be overcome. Nurses can shape the future of healthcare, ensuring that AI technologies are used ethically and effectively to enhance patients by addressing these challenges and leveraging AI's potential.

## AUTHOR CONTRIBUTIONS

*Conceptualization and methodology*: Abdulqadir J. Nashwan. *Writing—original draft, Writing—review & editing*: Abdulqadir J. Nashwan, J. C. A. Cabrega, Mutaz I. Othman, Mahmoud A. Khedr, Rami Naif, and Ahmad A. Mousa.

## CONFLICT OF INTEREST STATEMENT

The authors have no conflicts of interest to disclose.

## FUNDING INFORMATION

Open Access funding is provided by the Qatar National Library.

## Supporting information




Supporting Information
