# Supplementary figures and images for: The evolving role of nursing informatics in the era of artificial intelligence
Source: Int Nurs Rev. 2025 Jan 10;72(1):e13084. doi: 10.1111/inr.13084 (PMC11723855; doi:10.1111/inr.13084)

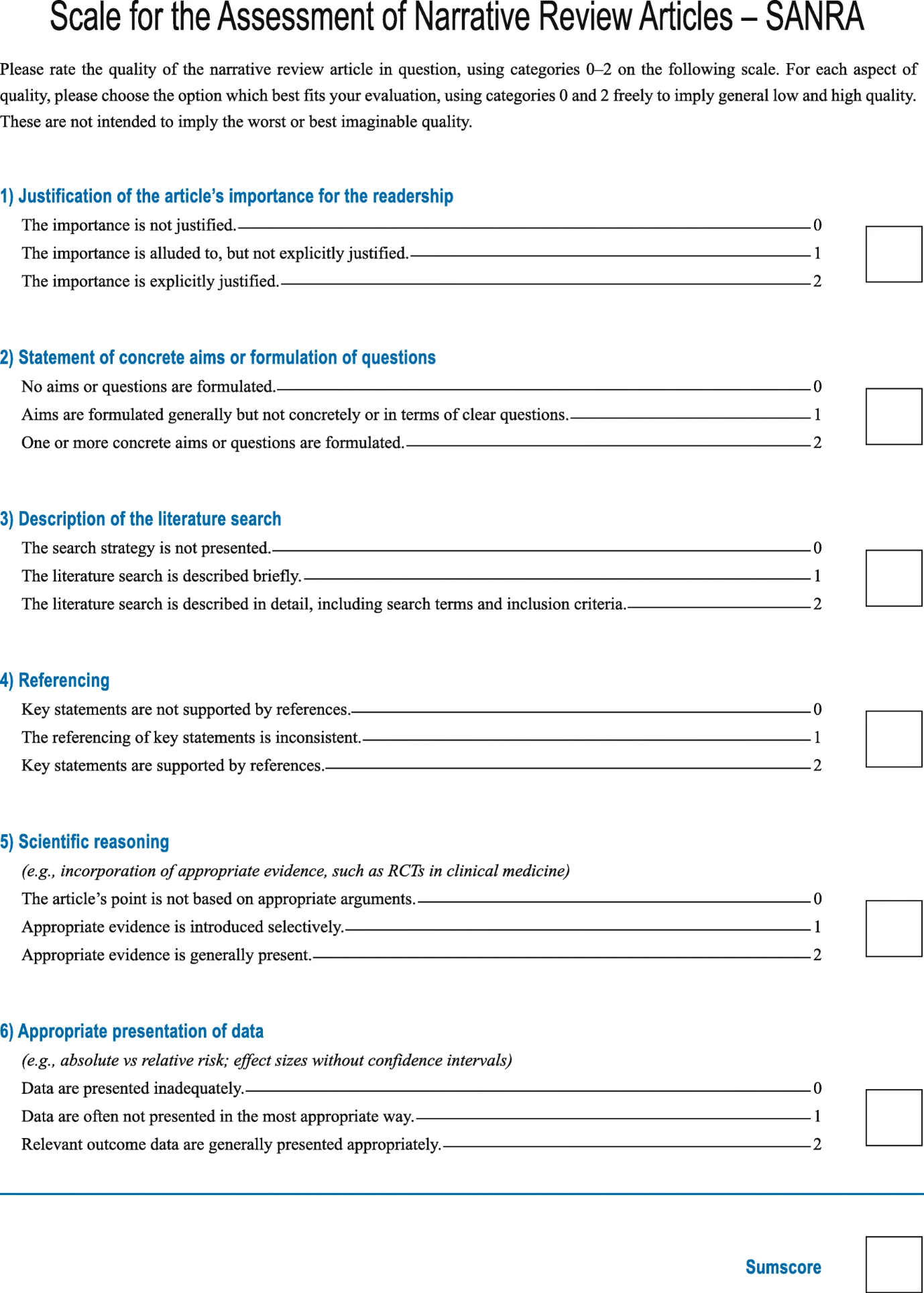


12

2

2

2

2

2

2

Supplement: Supplementary file 1 — Supporting Information [file INR-72-0-s001.docx]
